# Glimpses of the molecular mechanisms of β_2_-microglobulin fibril formation in vitro: Aggregation on a complex energy landscape

**DOI:** 10.1016/j.febslet.2009.05.005

**Published:** 2009-08-20

**Authors:** Geoffrey W. Platt, Sheena E. Radford

**Affiliations:** Astbury Centre for Structural Molecular Biology and Institute of Molecular and Cellular Biology, University of Leeds, Leeds LS2 9JT, United Kingdom

**Keywords:** DRA, dialysis-related amyloidosis, NMR, nuclear magnetic resonance, SEC, size exclusion chromatography, β_2_-microglobulin, Amyloid fibrils, Mechanisms, Structure

## Abstract

β_2_-microglobulin (β_2_m) is a 99-residue protein that aggregates to form amyloid fibrils in dialysis-related amyloidosis. The protein provides a powerful model for exploration of the structural molecular mechanisms of fibril formation from a full-length protein in vitro. Fibrils have been assembled from β_2_m under both low pH conditions, where the precursor is disordered, and at neutral pH where the protein is initially natively folded. Here we discuss the roles of sequence and structure in amyloid formation, the current understanding of the structural mechanisms of the early stages of aggregation of β_2_m at both low and neutral pH, and the common and distinct features of these assembly pathways.

## 1 Introduction

**Fig. 1:**
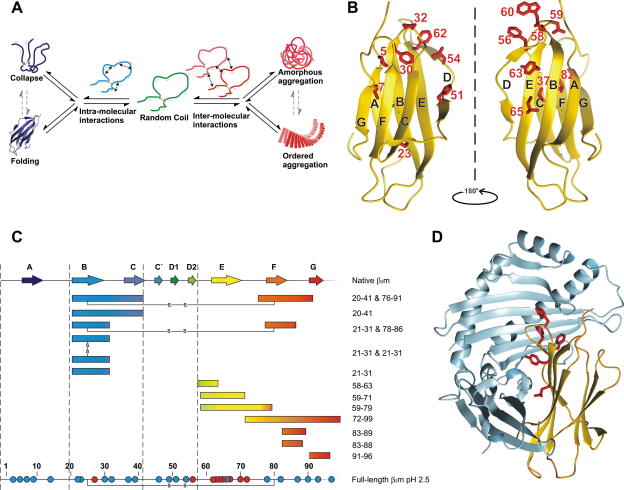
(A) Schematic illustration of the competition between intra-molecular and inter-molecular interactions in protein folding and assembly. When intra-molecular interactions prevail, proteins fold or form collapsed states (left side). By contrast, when inter-molecular interactions dominate, protein aggregation results (right side). (B) Monomeric β_2_m with residues discussed herein highlighted. (C) Amyloid forming properties of the sequence of β_2_m when isolated as peptides [10–15], or in the context of the full-length protein (the latter at pH 2.5). Red circles indicate residues that affect fibril formation kinetics, blue circles indicate positions where changes had little or no effect on kinetics [19]. (D) Structure of β_2_m (gold) in the MHC-1 complex. Hydrophobic residues present in the β-strand E region of β_2_m (Phe56, Trp60, Tyr62, Tyr63 and Leu65) that contact the heavy chain of the MHC-1 are highlighted.

The aggregation of proteins into amyloid fibrils has been studied in detail for more than 50 years, yet elucidation of the exact structural mechanisms of this process still provides a considerable challenge [1]. The heterogeneous nature of the protein aggregation landscape contributes to the complexity of this problem. Indeed, even at the earliest stage of this process the monomeric precursors of amyloid formation can be found as a complex mixture that can include natively folded, partially folded and highly unfolded protein species, any one of which could initiate the aggregation process. Furthermore, since the inter- and intra-molecular interactions that are involved in aggregation and protein folding are similar, much work remains to improve our understanding of the competition between these events, especially at the initial stages of amyloid formation (Fig. 1A) [2]. Moreover, multiple practical challenges exist as the formation of fibrils is stochastic and many of the species populated en route to the fibril product are unstable and only transiently formed. Understanding the biophysical nature of amyloid formation, however, is an important goal, not only because a number of diseases involve the deposition of amyloid fibrils [3], but also because the ability to form amyloid is known to be a fundamental property of all polypeptide chains under the appropriate conditions, independent of sequence [4]. However, the manner in which the generic cross-β structure of amyloid is accommodated by the differing sequences of proteins within their fibrillar architecture is not yet understood.

To comprehend the process of amyloid fibril formation in atomistic detail much work has been performed on a range of proteins in vitro. Here, we review some of the techniques employed and the results obtained in the study of the structural and molecular mechanisms of fibril formation of the protein β_2_-microglobulin (β_2_m). This protein folds natively into a β-sandwich fold consisting of two β-sheets, one containing four strands (ABED) and the other three (CFG) (Fig. 1B) [5]. A disulphide bond between two cysteines (residues 25 and 80) covalently links these sheets [5]. Intact wild-type β_2_m is the major component of amyloid fibrils deposited in the joints of patients on long-term haemodialysis, in a condition known as dialysis-related amyloidosis (DRA). In vivo, β_2_m is the non-covalently attached light chain of the human major histocompatibility complex 1 (MHC1) [5]. Naturally, β_2_m is shed into the serum and degraded by the kidneys, maintaining the serum β_2_m concentrations at 0.09–0.17 μM in healthy individuals [6]. However, in patients with renal failure the haemodialysis membrane is unable to remove β_2_m, resulting in increases in the serum concentration by up to 60-fold [7]. This rise in concentration alone is deemed not to be sufficient for fibril formation [8]. Thus other, as yet unknown, contributing factors lead to deposition of the full-length β_2_m as amyloid fibres in the synovium of joints.

Here we consider the current state of research into the mechanism of β_2_m fibril formation in vitro. We discuss the fibril formation pathways under different conditions, how they may be related to one another and to the mechanism occurring in vivo; and how the amyloid forming potential of β_2_m is modulated by sequence and structural changes.

## 2 Sequence versus structural determinants of fibril formation

Studies using prediction algorithms and peptide models have indicated that ∼60% of the sequence of β_2_m is highly amyloidogenic (Fig. 1C) [9–15]. However, the innate amyloid forming potential of the polypeptide sequence of β_2_m is modulated by structure, as the natively folded protein is impervious to aggregation [8,16]. At least a partial unfolding event must therefore take place in vivo to allow exposure of one or more aggregation-prone region(s) of the sequence to initiate amyloid formation. To study fibril formation of β_2_m in vitro, many destabilising conditions have been applied, by adding co-solvents, detergents or denaturants at neutral pH or by reducing the pH to drive the aggregation process on a biophysically feasible timescale [17].

At low pH (<pH 3.0), low ionic strength (⩽50 mM) and encouraged by agitation, β_2_m spontaneously aggregates in vitro to form fibrils with all of the hallmarks of amyloid [16]. Kinetic analyses of fibril growth coupled with a mutational screen have indicated that a single region approximately 10 residues in length (∼60–70) is important for determining the rate of fibril nucleation and elongation of the full-length protein under these conditions (Fig. 1C) [18,19]. This region of the sequence is enriched in aromatic residues and is predicted to be highly aggregation-prone by amyloid algorithms [9]. Other studies of the full-length β_2_m sequence at low pH have indicated that its ability to form amyloid is related to the stability of the fibrils and that introduction of the β-sheet breaking amino acid, Pro, in place of certain residues, especially Leu23, His51 or Val82, causes a reduction in fibril elongation kinetics [20]. Comparison of the effects of sequence alteration on the fibril growth kinetics of the intact, oxidised protein at low pH with the results of peptide studies is striking. While fragments corresponding to three regions (residues 20–40, 60–70 and ∼80–99) all form amyloid in isolation [10–15,19], in the context of the full-length protein chain mutation of residues in only one region (∼60–70) alters the fibril formation kinetics (Fig. 1C) [18,19]. One explanation of this observation is that structure in the initial denatured state may modulate the amyloid potential of the polypeptide chain, even when the initial amyloid precursor is highly unfolded (Fig. 1A) [19]. Indeed, nuclear magnetic resonance (NMR) analysis indicates that the acid unfolded state of β_2_m contains non-native residual structure, stabilised by the disulphide bond, which involves the clustering of hydrophobic residues in two regions (29–51 and 58–79) [21]. Therefore, even in this highly dynamic species the amyloid forming potential of the sequence may be modulated by structure, with the result that only one short stretch of residues (∼60–70) determines the aggregation potential of the entire 99-residue sequence. Examination of the crystal structure of β_2_m in the MHC class I complex illustrates why the protein may contain such a highly aggregation-prone sequence. Aromatic residues including Phe56, Trp60, Phe62 and Tyr63 (as well as the hydrophobic residue Leu65), which all lie in the aggregation-determining sequence, make important contacts with the MHC I heavy chain (Fig. 1D) [5]. Indeed, mutation of Trp60 to Gly results in a weakened interaction with the heavy chain, and a more stable native state that is less aggregation-prone [22]. This part of the sequence thus appears to have evolved for functional reasons (maintenance of the immune system) without consideration of the consequences for aggregation.

## 3 Structural mechanisms of the early events in β_2_m aggregation at pH 2.5

**Fig. 2:**
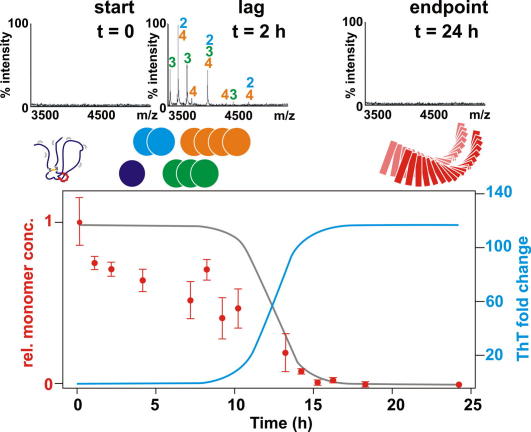
Species formed en route to amyloid fibrils by β_2_m at pH 2.5 as monitored by mass spectrometry and ThT fluorescence. During the lag phase (blue line, bottom figure), the concentration of monomeric protein (red circles) decreases more rapidly than expected based on ThT data (grey line). ESI-MS data (top) shows the presence of dimers, trimers and tetramers as well as monomers, but no larger species, during the lag phase. At the conclusion of the reaction no oligomers remain. Data were taken from Smith et al. [24].

At low pH β_2_m fibril formation proceeds rapidly from a highly dynamic state with lag-dependent kinetics [16,23], indicative of a nucleation-dependent mechanism. Mass spectrometry data indicates that small oligomers including dimers, trimers and tetramers (but no larger assemblies) are observed to form during the lag time of the aggregation process (Fig. 2) [24]. That higher order assemblies are not detected implies that larger oligomers may rapidly aggregate into fibrils, or such species may not be detectable by mass spectrometry. Numerical modelling studies of the kinetics of fibril formation at different protein concentrations, which included over 20 different possible assembly mechanisms, suggest that the initial molecular recognition events involve the assembly of monomers leading to the formation of a high-energy structural nucleus consisting of six β_2_m polypeptide chains. Elongation then proceeds rapidly to form amyloid-like fibres by monomer addition [23]. Structural detail of the observed oligomers is sparse but little ordered secondary structure is observed by FTIR [25], and these species do not bind the antibody A11 (unpublished data), which has commonly been used to detect toxic oligomers that form en route to amyloid fibrils from many different proteins [26]. Presently, the structural role of the aromatic-rich region involving residues ∼60–70 in these early assembly intermediates is unknown [18,19], although previous studies have noted the importance of aromatic stacking interactions in amyloid formation [27].

Information towards understanding the elongation of β_2_m fibrils was gained in a study at pH 2.5 by total internal reflection fluorescence microscopy, which indicated that this process is unidirectional when the fibrils are fixed on a glass surface, implying a polarised aggregation mechanism [28]. Interestingly, this method also indicated that fibrils elongate at a range of rates suggesting a variety of structural differences in the long-straight amyloid structures formed at pH 2.5 and highlighting the complexities of studying amyloid fibril formation whereby different fibril structures co-exist at equilibrium that are difficult to discern without recourse to detailed structural analysis.

## 4 A structural trigger at neutral pH?

**Fig. 3:**
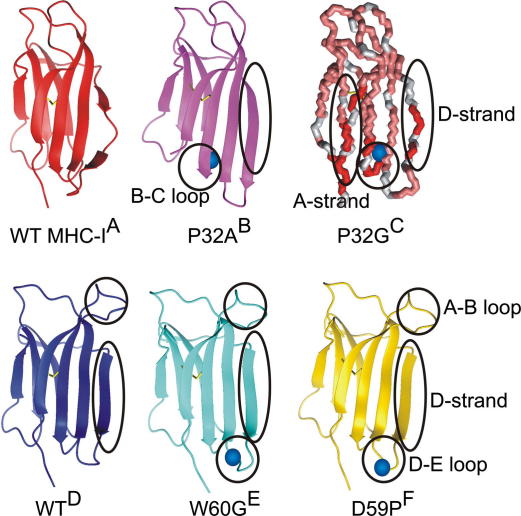
Structures of wild-type β_2_m and several variants mentioned herein, blue spheres show the site of mutation where relevant. Regions of major conformational change are highlighted. (A) WT β_2_m in the MHC-1 complex (PDB: 1DUZ) [5]. (B) P32A variant of β_2_m that crystallises as a dimer and forms digomers in the presence of Cu^2+^ (2F8O) [31]. (C) Model of structural changes in a folding intermediate of β_2_m, populated at high levels in the P32G variant, mapped onto the NMR structure of wild-type β_2_m. Regions that are most perturbed in comparison with wild-type β_2_m are shown in red, those that show little perturbation are pink and no information is available for those in grey [37]. (D) X-ray crystallographic structure of a rare monomeric species of wild-type β_2_m with a straight β-strand D (1LDS) [44]. (E) W60G variant of β_2_m that has a decreased amyloid propensity (2Z9T) [22]. (F) D59P β_2_m displays an increased propensity to form amyloid (3DHJ) [38].

To mimic conditions that are more physiologically relevant, studies in vitro have adopted a number of methods to drive amyloid formation of β_2_m at neutral pH, since the protein is initially natively folded at pH 7. Biological components, including glycosaminoglycans, proteoglycans, collagen and Cu^2+^ (the latter in the presence of 1 M urea) have all been used to promote, or induce, fibril formation at neutral pH in vitro [8,29–31]. In addition, co-solvents such as TFE or SDS, alteration of the physical environment by ultrasonication, elevated temperature or stirring at high concentrations of salt induce fibril formation from wild-type β_2_m at, or close to, neutral pH [32–35]. Alterations of the protein sequence have also been used to stimulate the formation of fibrils at neutral pH. Specifically, truncation of six residues from the N-terminal region (ΔN6), as well as mutation in this region (P5G) or in the B/C or D/E loops (P32A, P32G, D59P) of the protein all enhance its ability to form amyloid in vitro (Fig. 3), while substitutions elsewhere in the protein have little effect [31,36–38]. These studies have the common feature that they encourage partial unfolding of β_2_m, allowing the aggregation-prone regions of the polypeptide sequence to be exposed and to participate in intermolecular interactions.

The β_2_m variants, P32G and P32V, have been used to show that wild-type β_2_m populates a native-like folding intermediate (called I_T_) en route to the native state that contains a non-native *trans* Pro32 bond [37,39,40]. Increased population of this intermediate in the variant P32G was shown to be concomitant with increased ability of the protein to elongate β_2_m fibril seeds [37]. Interestingly, it may be that a specific *trans* Pro32 residue is important for β_2_m to nucleate amyloid fibrils at neutral pH, as both P32G and P32V β_2_m which form *trans* Gly32 or Val32 cannot form amyloid-like fibrils spontaneously, although P32G can elongate stabilised seeds more efficiently than wild-type β_2_m under the conditions employed [37,40]. Other variants, such as P5G and ΔN6, also affect isomerisation of the Pro32 peptide bond, promoting population of I_T_ and enabling fibril nucleation at pH 7.0 [36]. In separate studies β_2_m has been shown to form oligomers and fibrils at neutral pH by addition of Cu^2+^ and 1 M urea [8,31]. This induces the formation of a non-native species, called M^∗^, that appears identical to I_T_ by size exclusion chromatography (SEC) [36]. Coordination of the metal ion promotes peptide bond isomerisation at Pro32 [31], and the subsequent rapid formation of oligomers as judged by SEC, providing further supporting evidence that the isomerisation of Pro32 is a key initial step on the pathway to fibrils [8,31,36].

Structural studies of the trapped folding intermediate, I_T_ (or M^∗^), have provided molecular insights into the possible aggregation mechanisms of β_2_m at neutral pH. Burst-phase amplitudes from real-time NMR studies of β_2_m folding indicate that residues distal to Pro32 maintain native-like structure in the intermediate, while regions proximal to Pro32 in the B/C loop are disordered until *trans* to *cis* isomerisation of the Pro32 peptide bond takes place [40]. Consistent with this, studies of P32G by NMR have shown that residues in the B/C loop, the adjacent F/G loop and the edge β-strands A and D are altered in the intermediate I_T_ (Fig. 3). Crystallographic data for a mutant, P32A, that populates the native-like M^∗^ state at neutral pH, shows that this conformer results in substantial reorganisation of aromatic side-chains that are present in the core of the native protein [31]. In this structure, a number of aromatic and hydrophobic residues are displaced including Phe30, Phe56, Trp60, Phe62, Tyr63 and Leu54. These rearrangements present a strip of exposed hydrophobic residues on the protein surface, providing a possible avenue for protein aggregation. Furthermore, the structural changes lead to alterations in the organisation of residues 52–56, including abolition of the β-bulge that is present in the native β-strand D and is a deterrent to edge strand aggregation (Fig. 3) [41]. This species crystallises as a dimer underlining its potential to form intermolecular interactions [31].

Other regions of the β_2_m sequence have been proposed to modulate fibril formation at neutral pH and do not involve isomerisation of the Pro32 peptide bond. Substitution of Trp60 in the D/E loop with Gly increases the stability of the native state and reduces the ability of the protein to aggregate at neutral pH in the presence of TFE [22]. Removal of this large, hydrophobic side chain will not only reduce the inherent aggregation potential of the protein chain but, by decreasing strain in the loop region adjacent to the aromatic-rich, amyloidogenic sequence of β-strand E [18], the native state is stabilised, hence further reducing its ability to aggregate (Fig. 3). Indeed, a D59P variant that increases strain in the D/E loop region and reduces protein stability shows an increased potential to form fibrils (Fig. 3) [38].

## 5 A common fibrillar architecture is formed from diverse precursors

**Fig. 4:**
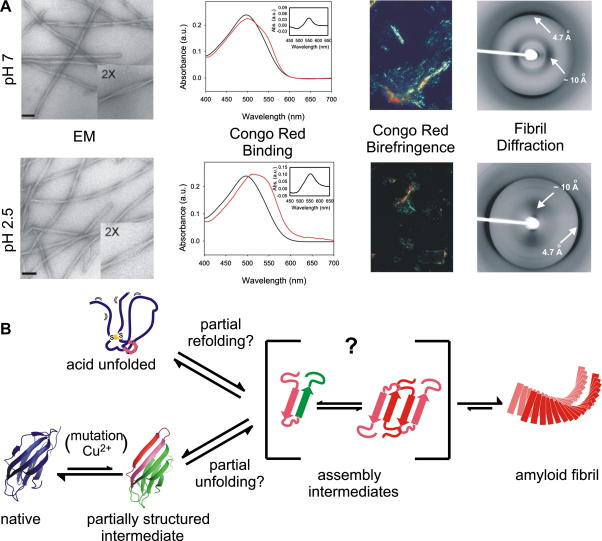
(A) Comparison of experimental data for fibrils of β_2_m formed at pH 7.0 and 2.5. EM scale bar indicates 100 nm. Absorbance spectra of Congo Red free in solution (black line) and bound to β_2_m fibrils (red line) as well as the difference spectra (inset). Both fibril types give rise to red-green birefringence in the presence of Congo Red. The X-ray fibre diffraction patterns show reflections at 4.7 Å and ∼10 Å, consistent with a cross-β structure. Data were taken from Jahn [50]. (B) Scheme for convergence of the mechanisms of fibril formation at pH 2.5 and 7.0. Regions with high amyloidogenic propensity are displayed in pink. It is not known precisely how oligomers stack or whether they have ordered β-sheet, however, increased intermolecular protein–protein interactions (red) may be important in the reaction pathway.

Akin to a protein folding landscape, aggregation into amyloid may be achieved via a number of routes. In aqueous conditions at pH 2.5 and 7.0 amyloid-like fibrils are formed from β_2_m that show a long-straight, left-hand twisted and unbranched morphology when observed by EM and AFM (Fig. 4A) [42]. Fibrils formed at both pH values bind the amyloid-specific dyes ThT and Congo Red; display characteristic cross-β structure as evidenced by X-ray fibre diffraction (Fig. 4A) and exhibit an identical β-sheet content when studied by FTIR [42]. However, some differences between the fibrils must exist as those formed at pH 2.5 depolymerise at pH 7 unless stabilised by co-factors such as glycosaminoglycans [29]. Comparison of the fibrils formed at pH 2.5 and 7.0 by FTIR, a technique that is highly sensitive to the length and twist of β-strands, suggests that the polypeptide chain is ordered in a similar parallel fashion in both fibril types [25,42]. These structural similarities are shared by ex vivo fibrils obtained from DRA patients, implying that study of the processes that lead to fibrils in vitro under aqueous conditions, even at low pH, may provide relevant information about the formation of fibrils in disease. These findings beg the question of how similar the assembly pathways are and whether these separate pathways converge to form a common fibrillar product (Fig. 4B). Interestingly, however, the fibrils produced in the presence of SDS or TFE show distinct morphological properties, compared with those formed in the absence of these additives at neutral pH, suggesting that alternative conformations of the fibres (polymorphs) can be formed by fine tuning of the solution conditions [32,43].

## 6 Progress towards a unifying mechanism of β_2_m fibril formation

One of the most striking observations of the β_2_m assembly pathways is that the fibrils formed commencing from a highly denatured state or a native-like precursor are apparently indistinguishable, suggesting that their assembly pathways must converge to a similar fibrillar product. This could occur by unfolding of native β_2_m to allow reorganisation of the protein structure; or by refolding of the highly dynamic polypeptide chain at pH 2.5 to a more structurally ordered intermediate species (Fig. 4B). The kinetics of fibril formation suggest that the acid unfolded precursor can readily access a critical aggregation-prone species, since amyloid formation proceeds rapidly (within hours) under these conditions [18,23]. By contrast, at pH 7, even in the presence of fibril seeds, fibril formation takes weeks [36]. Similarities between the assembly mechanisms are supported by the proposal that aromatic interactions are crucial for driving fibrillogenesis under both sets of conditions [12,18,19,31]. Indeed, the dimeric structure of M^∗^ [31], indicates that interactions between specific and structurally organised hydrophobic and aromatic residues may lead to fibrillation. Moreover, removal of the β-bulge from β-strand D, as observed for many of the fibril-forming variants of β_2_m formed at neutral pH (Fig. 3), could provide a site for productive intermolecular edge strand interactions. Interestingly, a rare conformer of wild-type β_2_m with a straightened β-strand D has been observed in crystallography studies (Fig. 3) [44]. Whilst this wild-type conformer has different sidechain organisation to that observed for the variants capable of forming fibrils at neutral pH (P32A, D59P) it provides evidence (alongside that of other variants, such as W60G) [22,43] that this edge strand straightening is not sufficient to promote fibril growth. Another convergent feature arises from a suggested model for the fibrils formed at neutral pH that contains a highly charged surface [31], which could be neutralised at low pH, perhaps allowing for a convergence of the mechanisms of assembly at acidic and neutral pH and explaining why the kinetics of fibril formation are much more rapid under acidic conditions. An additional common characteristic is that many variants capable of fibril formation at neutral pH have the effect of destabilising the N-terminal region of the protein [31,37,40], which is also highly unfolded in the structural ensembles formed at low pH [21]. The disruption of this region has been proposed as key to amyloid formation [45]. Indeed, the double variant, P32G/I7A that combines a *trans* peptide bond at Pro32 with destabilisation of the N-terminal region forms fibrils spontaneously at neutral pH [42]. The role of *trans* Pro32 in fibril formation at low pH is currently unknown, although ∼80% of the molecules would be expected to contain the *trans* conformation in the acid denatured state [46]. Moreover, the observation that the rate of fibril formation of P32G is similar to that of wild-type β_2_m when studied at pH 2.5 is suggestive of a common *trans* amyloid precursor [19].

The possibility that partial or global unfolding of the initially folded monomer of β_2_m is required for fibrillogenesis at neutral pH is supported by data from a variant of β_2_m that is cleaved at Lys58 (ΔK58), adjacent to the aromatic-rich, aggregation-prone region (∼60–70), and retains a native fold in vitro [47]. This variant is fibrillation-prone under conditions similar to those found in vivo (<pH 6.8), where it more frequently populates a highly unprotected state [48]. The aggregation-competent species formed in these studies must, however, contain some specific structural attributes as simply creating a variant of β_2_m that is highly unstable and frequently visits an unfolded state (such as V37A) does not engender amyloidogenicity alone [49].

## 7 Summary and outlook

Studies of the molecular mechanisms of β_2_m aggregation both under denaturing and native conditions have provided important new insights into the structural changes associated with fibril formation for this protein that may have relevance for understanding amyloid in general. The sequence of β_2_m has a high amyloid forming potential as inferred from aggregation prediction algorithms and peptide studies [9–15]. However, work on the full-length protein indicates that the aggregation potential of this sequence is highly modulated by structure, not just in the native state, wherein the aggregation-prone regions are completely sequestered in the native core, but also in the highly dynamic acid unfolded state. Thus, whilst peptide fragment studies are of great utility in answering fundamental questions regarding amyloid formation, their study in context of full-length proteins can be misleading.

The fact that many different proteins with unrelated sequences and native folds can all form amyloid-like fibrils with nucleation-dependent kinetics implies the possibility of a common mechanism. The finding that β_2_m can form similar long-straight amyloid-like fibrils commencing from two very different structural states reinforces the view of a generic assembly mechanism. Under each set of conditions similar hydrophobic and aromatic regions of the sequence appear to be key to the aggregation mechanism. While reducing the stability of the native fold and increasing global or subglobal unfolding events is important in initiating amyloid formation, some structural preference, for instance the presence of a non-native *trans* Pro32, is clearly linked to the ability to aggregate. Even in the acid unfolded state it appears that specifically structured species presenting aggregation-prone regions are required for amyloid formation, whilst other transiently structured regions may modulate aggregation by sequestering sequences with high amyloid forming potential.

It is currently unclear whether the same oligomeric species are populated during assembly at acidic and at neutral pH and future endeavours will need to focus on the structural properties of the oligomers that form as aggregation proceeds. Such studies may provide clues as to how fibrillation can be impeded therapeutically and how fibril formation proceeds mechanistically. Indeed, whilst oligomers are commonly observed during fibril formation, there is as yet no definitive proof that such species are on pathway to the fibrillar form. Furthermore, atomistic insights into the structure of β_2_m amyloid fibrils will be required to provide unequivocal evidence for the convergence of the assembly pathways at pH 2.5 and 7.0 and to provide detailed information as to whether the protein is completely refolded in the fibril structure or retains remnants of its native fold. Towards this goal recent cryo-EM studies have shown a remarkably complex architecture for the fibrils formed from β_2_m at pH 2.5 [51]. Ultimately, as the studies on β_2_m have helped to confirm, the amyloid-forming potential of a protein is clearly a function of how structure modulates the intrinsic β-aggregation propensity of the amino acid sequence, not just for the natively folded protein but also for proteins that aggregate from highly unfolded initial precursor states.
